# A Case of Posterior Reversible Encephalopathy Syndrome Complicating a Thoracic Spinal Subdural Hematoma

**DOI:** 10.7759/cureus.101703

**Published:** 2026-01-16

**Authors:** Tamotsu Gotou, Yuto Munemura, Takahiro Hagihara, Yamato Wada, Kyoji Hashimoto

**Affiliations:** 1 Department of Emergency and Critical Care Medicine, Tottori Prefectural Central Hospital, Tottori, JPN; 2 Department of Pediatric Emergency and Critical Care Medicine, Tottori Prefectural Central Hospital, Tottori, JPN

**Keywords:** hypertensive crisis, mri- magnetic resonance imaging, posterior reversible encephalopathy syndrome (pres), spinal subdural hematoma, thoracic spine

## Abstract

Posterior reversible encephalopathy syndrome (PRES) is a clinicoradiological syndrome characterized by acute neurological symptoms such as headache, seizures, altered consciousness, and visual disturbances, along with neuroimaging findings that typically demonstrate vasogenic edema predominantly involving the parieto-occipital regions. Common precipitants include hypertension, renal dysfunction, hypertensive disorders of pregnancy, immunosuppressive agents, and perioperative factors; however, PRES in association with spinal hematoma has rarely been reported.

A 68-year-old man developed acute back pain while stretching and presented to the emergency department. On initial evaluation, no focal neurological deficits were evident, and he was discharged after adequate analgesia. The following day, he returned with recurrent back pain. Thoracic spine magnetic resonance imaging (MRI) demonstrated findings suggestive of a spinal subdural hematoma at T3/4-T5/6, necessitating further inpatient evaluation. After admission, he experienced persistent intense pain accompanied by marked hypertension, with a peak blood pressure of 230/140 mmHg. He subsequently developed generalized seizures associated with headache and visual disturbances. Brain MRI revealed hyperintensity on T2-weighted and fluid-attenuated inversion recovery (FLAIR) images predominantly in the bilateral parieto-occipital regions, consistent with PRES. In the intensive care unit, blood pressure was controlled with continuous nicardipine infusion, targeting a systolic blood pressure of ≤160 mmHg and carefully titrating the infusion to avoid a decrease of >50 mmHg within the first hour, and analgesia/sedation was provided with fentanyl and dexmedetomidine, with subsequent resolution of neurological symptoms. He was later transitioned to oral antihypertensive and analgesic medications, with improvement on follow-up imaging. Although PRES complicating spinal hematoma is uncommon, several potential PRES triggers may be involved, including pain-related blood pressure fluctuations, sustained hypertension, renal and fluid balance disturbances, autonomic influences, perioperative factors, cerebrospinal fluid leakage, and cerebral vasospasm. When hypertension and new neurological symptoms, such as headache, visual disturbances, or seizures, occur during the clinical course of spinal hematoma, PRES may be considered, and prompt blood pressure control with brain MRI evaluation may be warranted.

## Introduction

Posterior reversible encephalopathy syndrome (PRES) is a clinicoradiological syndrome characterized by acute neurological symptoms and distinctive brain magnetic resonance imaging (MRI) findings, most commonly vasogenic edema with a predominant parieto-occipital distribution [1]. In previous reports, PRES has also been termed reversible posterior leukoencephalopathy syndrome (RPLS). PRES is multifactorial and has been described in a wide range of clinical settings, including hypertensive emergencies, renal failure, hypertensive disorders of pregnancy, autoimmune diseases, infections, exposure to immunosuppressive agents or chemotherapy, and perioperative periods [1-3]. Proposed mechanisms include cerebral hyperperfusion and blood-brain barrier disruption due to failure of cerebral autoregulation during rapid blood pressure elevation, as well as pathways centered on endothelial dysfunction and vasoconstriction [2-3].

Spinal hematoma (epidural or subdural) is an urgent spinal condition that can cause acute pain and neurological deficits due to spinal cord compression. However, reports describing the coexistence of spinal hematoma and PRES appear to be limited [4-8]. Here, we report a case of PRES developing during the course of a thoracic spinal subdural hematoma and discuss diagnostic and therapeutic considerations.

## Case presentation

A 68-year-old man with no relevant past medical history developed acute back pain while stretching and presented to the emergency department. No focal neurological deficits were evident on initial assessment, and he was discharged after his pain improved. The following day, his back pain recurred with nausea, and he returned to the hospital.

On arrival, he was alert. His vital signs were as follows: blood pressure at 192/60 mmHg, heart rate at 58 beats/min, SpO₂ at 99% on room air, and temperature at 36.5°C. The severity of his back pain was rated as 8 on the numerical rating scale (NRS). Neurological examination showed no apparent motor weakness, sensory deficit, or bladder/bowel dysfunction.

Laboratory testing demonstrated a minimal inflammatory response, with no abnormalities in coagulation parameters or renal function (Table 1). 

**Table 1:** Laboratory findings on admission Laboratory testing on admission demonstrated a minimal inflammatory response, with no abnormalities in coagulation parameters or renal function. WBC: white blood cell count; Hb: hemoglobin; Plt: platelet count; PT-INR: prothrombin time–international normalized ratio; CRP: C-reactive protein; T. Bil: total bilirubin; AST: aspartate aminotransferase; ALT: alanine aminotransferase; LDH: lactate dehydrogenase; CK: creatine kinase; BUN: blood urea nitrogen.

Parameter	Result	Unit	Reference range
WBC	8.9	×10^3/µL	3.3-8.6
Hb	15	g/dL	13.7-16.8
Plt	14	×10^4/µL	15.8-34.8
PT-INR	1.0	-	0.9-1.1
D-dimer	1.1	µg/mL	≤1.0
CRP	0.9	mg/dL	≤0.14
T. Bil	1.7	mg/dL	0.4-1.5
AST	23	IU/L	13-30
ALT	17	IU/L	10-42
LDH	211	IU/L	124-222
CK	117	U/L	59-248
BUN	19	mg/dL	8-20
Creatinine	0.6	mg/dL	0.65-1.07
Sodium	138	mmol/L	138-145
Potassium	3.5	mmol/L	3.6-4.8
Chloride	109	mmol/L	101-108
Calcium	9.0	mg/dL	8.8-10.1

**Figure 1:**
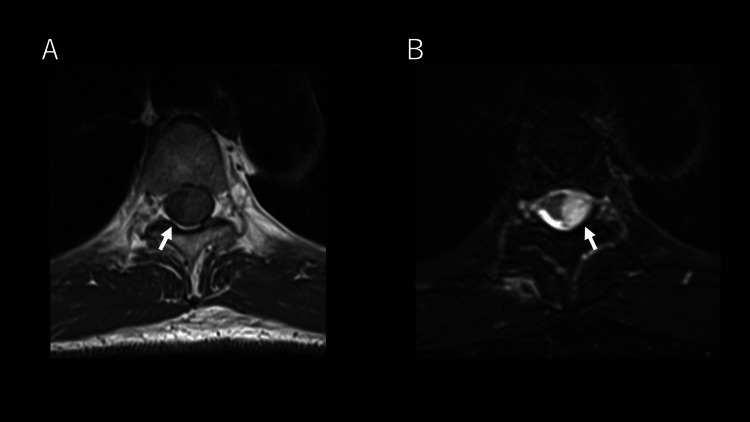
Thoracic spine MRI on the day of admission demonstrated findings suggestive of a spinal subdural hematoma extending from T3/4 to T5/6 (A) T1-weighted image shows no displacement or alteration of the epidural fat, supporting a subdural rather than an epidural lesion. (B) T2-weighted image demonstrates a hyperintense mass-like lesion compressing the spinal cord. Note: The left side of each image corresponds to the patient’s right side. MRI: Magnetic resonance imaging

An MRI of the thoracic spine revealed findings suggestive of a spinal subdural hematoma at the T3/4-T5/6 levels. No displacement of epidural fat was noted, supporting a subdural rather than an epidural lesion (Figure 1). The absence of antecedent trauma, invasive procedures, or antithrombotic therapy supported the diagnosis of spontaneous thoracic spinal subdural hematoma.

**Figure 2:**
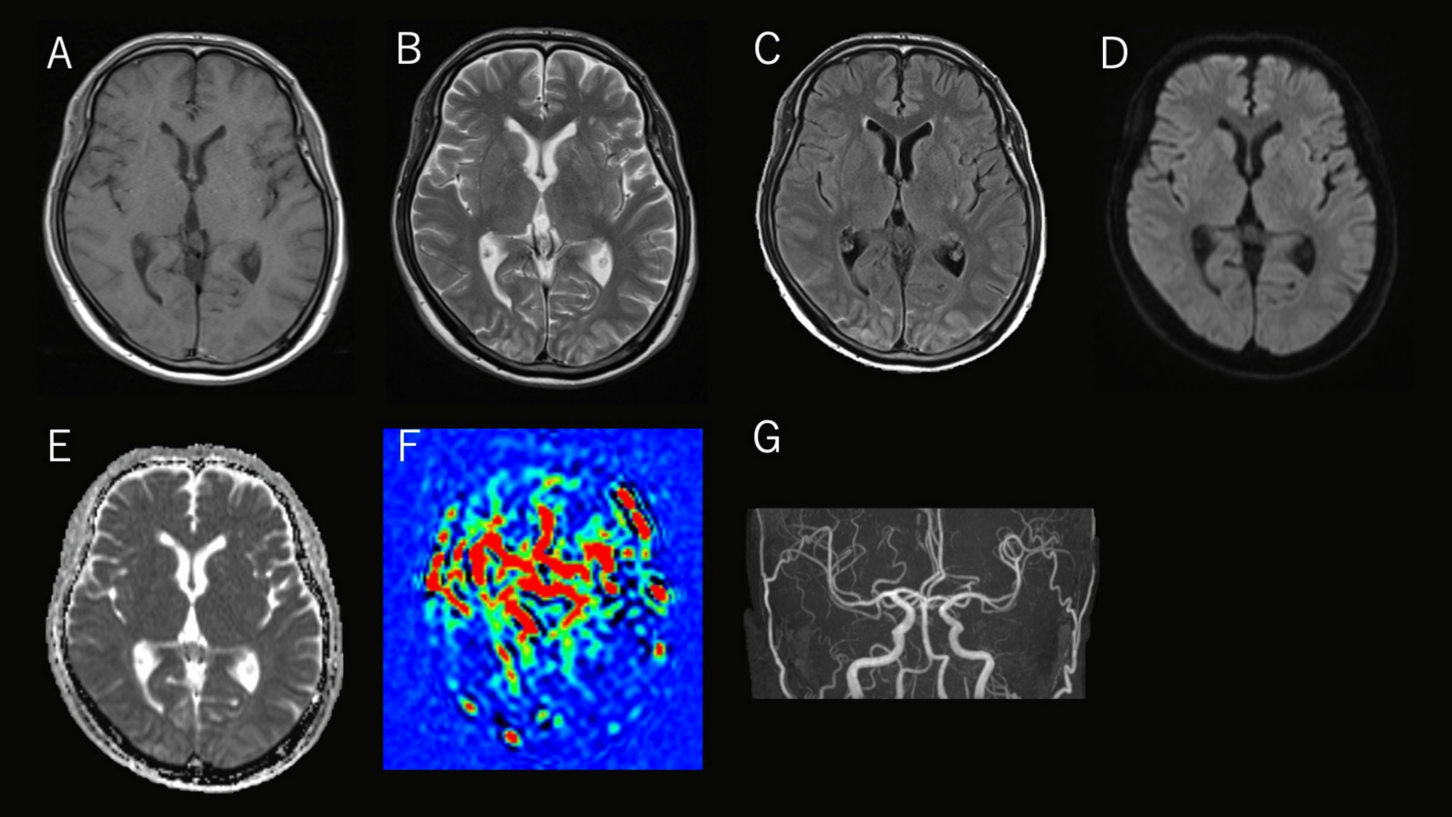
Brain MRI findings on the day of admission (A) T1-weighted images show no abnormal signal intensity. (B) T2-weighted and (C) FLAIR images demonstrate hyperintensity involving the cortex and subcortical white matter, predominantly in the bilateral parieto-occipital regions. (D) Diffusion-weighted imaging (DWI) shows no corresponding hyperintensity, and (E) the apparent diffusion coefficient (ADC) map shows increased signal without ADC reduction, indicating the absence of true diffusion restriction. (F) Arterial spin labeling (ASL) demonstrates hyperperfusion in the bilateral middle cerebral artery (MCA) and posterior cerebral artery (PCA) territories. (G) Magnetic resonance angiography (MRA) shows increased visualization/prominence of the bilateral anterior cerebral artery (ACA), MCA, and PCA. Overall, these findings are consistent with posterior reversible encephalopathy syndrome (PRES). Note: The left side of each image corresponds to the patient’s right side.

The patient was managed conservatively with analgesia, bed rest, and oral antihypertensive therapy (amlodipine); despite this, hypertension persisted in association with refractory pain. After admission, he developed a headache and visual disturbances, followed by generalized seizures. After the seizure, his blood pressure increased to 230/140 mmHg, and his Glasgow Coma Scale (GCS) score was 11 (E4V2M5). Brain MRI revealed hyperintensity on T2-weighted and fluid-attenuated inversion recovery (FLAIR) images in the cortex and subcortical white matter, predominantly involving the bilateral occipital lobes. No hyperintensity was observed on diffusion-weighted imaging (DWI), and the lesions were hyperintense on the apparent diffusion coefficient (ADC) map, indicating the absence of diffusion restriction. Based on these findings, PRES was diagnosed (Figure 2).

**Figure 3:**
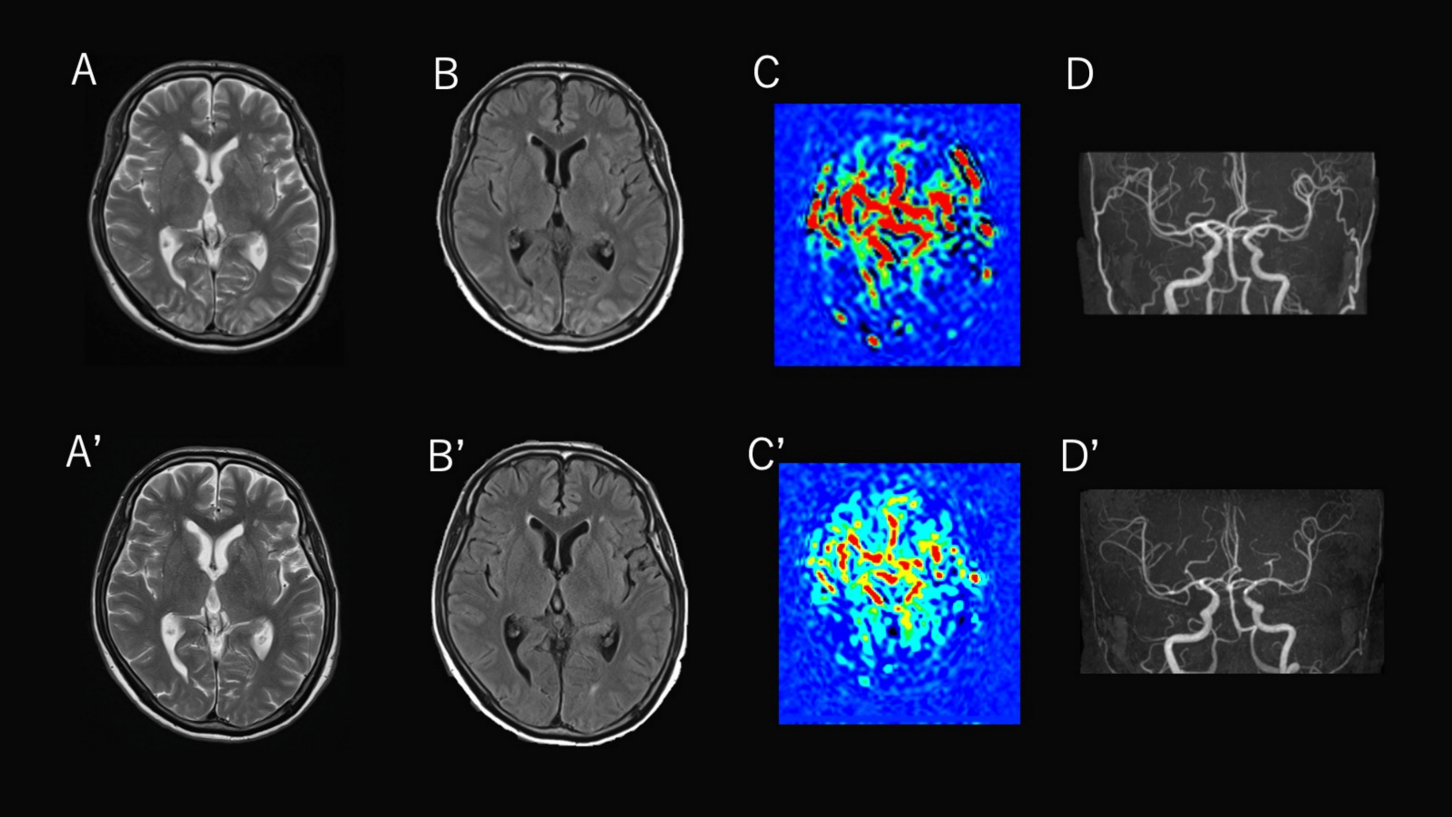
Comparison of brain MRI findings between day 1 and day 10 of hospitalization The upper row shows images obtained on day 1 of hospitalization (A–D), and the lower row shows images obtained on day 10 of hospitalization (A’–D’). T2-weighted (A, A’) and FLAIR (B, B’) images demonstrate that the occipital hyperintensity observed on day 1 of hospitalization resolved by day 10 of hospitalization. ASL (C, C’) shows that the hyperperfusion seen on day 1 of hospitalization disappeared on day 10 of hospitalization. MRA (D,D’) shows that the increased arterial visualization/prominence noted on day 1 of hospitalization also resolved by day 10 of hospitalization. Note: The left side of each image corresponds to the patient’s right side.

He was admitted to the intensive care unit (ICU), where blood pressure was gradually controlled with continuous nicardipine infusion, targeting a systolic blood pressure ≤160 mmHg and carefully titrating the infusion to avoid a decrease of >50 mmHg within the first hour. Fentanyl and dexmedetomidine were administered for analgesia and sedation. His neurological symptoms resolved promptly without recurrence. He was subsequently transitioned to oral antihypertensives (amlodipine and azilsartan) and oral analgesics (acetaminophen and celecoxib). A repeat MRI on hospital day 10 showed resolution of the signal abnormalities observed at presentation, and he was discharged on hospital day 15 (Figure 3). 

## Discussion

PRES is typically precipitated by acute or fluctuating hypertension and is radiologically characterized by predominantly posterior vasogenic edema. In this patient, PRES developed during conservative management of a thoracic spinal subdural hematoma (T3/4-T5/6), following refractory pain with marked hypertension (peak 230/140 mmHg) and subsequent headache, visual disturbance, and generalized seizures. The prompt clinical and radiological reversibility supports PRES as the unifying diagnosis [1-3].

Pain-related sympathetic activation is a plausible driver of rapid blood pressure elevation and variability, which may overwhelm cerebral autoregulation and promote endothelial dysfunction [2,3]. In the present case, sustained severe back pain preceded the hypertensive surge and neurological deterioration, while admission laboratory tests did not demonstrate overt renal dysfunction or coagulopathy (Table 1), making alternative systemic predispositions less prominent. In addition, the thoracic level of the lesion raises the possibility of autonomic dysregulation as a contributor to blood pressure instability, including autonomic dysreflexia (mass reflex) triggered by noxious stimuli.

Management of PRES centers on removal or mitigation of precipitating factors and careful blood pressure control, often requiring ICU-level monitoring in severe cases [9,10]. Although an optimal target for PRES has not been established, an often-cited approach in hypertensive emergencies is to avoid excessive early reductions (e.g., not exceeding approximately 25% within the first hour), followed by cautious normalization over 24-48 hours [10]. In our patient, blood pressure was gradually controlled with continuous nicardipine infusion while providing analgesia/sedation with fentanyl and dexmedetomidine; the target systolic blood pressure was ≤160 mmHg, and the infusion was titrated to avoid a decrease of >50 mmHg within the first hour. Clinical stabilization was followed by transition to oral therapy once stable [1-3].

At symptom onset, acute ischemic stroke and postictal imaging changes were considered. However, the absence of diffusion restriction on DWI/ADC and the posterior-predominant distribution, together with radiological resolution on follow-up imaging, favored PRES. Given the recognized overlap between PRES and reversible cerebral vasoconstriction syndrome (RCVS), vascular imaging may be useful when clinical features suggest RCVS. As intracranial hypotension or cerebrospinal fluid leak has also been described in association with spinal pathology, targeted evaluation can be considered when clinical or imaging findings raise suspicion. In the present case, brain MRI did not demonstrate typical features suggestive of intracranial hypotension (e.g., diffuse pachymeningeal enhancement or brain sagging).

This case underscores a practical diagnostic message: during spinal hematoma management, new-onset headache, visual symptoms, seizures, or altered consciousness in association with severe or fluctuating hypertension should prompt consideration of PRES and a timely brain MRI. An integrated strategy of adequate analgesia/sedation, careful hemodynamic reduction, and assessment of contributors such as autonomic dysregulation may facilitate rapid recognition and favorable neurological recovery.

## Conclusions

We report a case of PRES developing during conservative management of a thoracic spinal subdural hematoma, associated with marked hypertension accompanied by headache, visual disturbances, and seizures. Reports of PRES complicating spinal hematoma are limited, and cases associated with thoracic spinal subdural hematoma are rare. Multiple factors may be involved, including pain-related sympathetic activation and blood pressure spikes, sustained hypertension, fluid/electrolyte disturbances, renal function, perioperative factors, autonomic triggers, CSF leak, and cerebral vasospasm. During the clinical course of spinal hematoma, clinicians should consider assessing blood pressure trends and potential triggers in parallel with pain evaluation. If neurological symptoms (headache, visual disturbances, seizures, or altered consciousness) occur, evaluation for PRES, including brain MRI, may be considered along with prompt blood pressure management.
